# Degloving Injuries of Upper Extremity: A Strategy with Full Thickness Skin Mesh

**DOI:** 10.29252/wjps.7.3.372

**Published:** 2018-09

**Authors:** Christian Weinand

**Affiliations:** Clinic for Plastic and Aesthetic Surgery, Hand Surgery, Helios Clinics Gifhorn, University of Magdeburg, Germany

**Keywords:** Degloving, Injury, Extremity, Skin, Mesh, Graft, Defect

## Abstract

Degloving injuries of the upper extremity during work are rare nowadays, because of effective worker protection devices. However, these devastating injuries still occur today during motor vehicle car accidents and surgeons have to be aware of the possibilities of wound coverage for these large, contaminated wounds. We present two cases of degloving injuries of the hand and entire forearm using degloved skin as meshed full thickness skin graft to cover the entire wound. Two patients were admitted to our hospital, presenting large degloving injuries of the entire forearm and hands. Both patients sustained their injuries by being dragged by moving trains and presented additional fractures of the metacarpal bones and in both cases the little finger had to be amputated. The degloved skin was cleansed and meshed as a full thickness skin graft 1:3, using a Brennen Mesher. In both patients, complete wound coverage was achieved using the degloved skin as meshed full thickness skin graft. Attachment to the wound was achieved by vacuum closure device, however, ninety percent of the graft did attach. In both cases, shrinking of the full thickness skin grafts was noted. Both of them received physiotherapy and gained nearly complete function of the injured hand and wrist. When larger parts of skin are lost in degloving injuries meshing the degloved skin as full thickness skin grafts good wound coverage of larger, contaminated defects can be achieved.

## INTRODUCTION

Improved work safety measures have made degloving injuries of hand and forearm a rare occurrence. However, they still occur in road traffic accidents or during private activities.^1^ Ring-avulsion injuries, conveyer belt and road traffic related degloving injuries are nowadays seen more often.^2^ In most cases the musculoskeletal unit, the vascularity of the remnant tissues, and the length of the limb/fingers/toes are preserved. However, the underlying tendons, muscles and neurovascular bundles are exposed. Hence, the main goal is preservation of function and length by early reconstruction by replantation or revascularization.^1^^,^^3^

When the skin is totally removed from the body, it can be replaced by replantation. In the case which the skin is still attached to the body but has been physically degloved and nourishing vessels were damaged, it can be re-vascularized by either arterial or venous anastomosis or both.^4^^-^^6^ Thereby quality and color of skin can be maintained.^1^^,^^4^ However, there are some incidents when these options are no longer possible.^2^ One option is then to thin avulsed skin and use it over degloved parts as full thickness skin graft.^7^ Another possibility is to use degloved skin as a source for split thickness skin grafts^8^ or to cover denuded areas with pedicled flaps, flaps derived from the abdominal wall or free flaps.^2^^,^^9^^,^^10^ Amputation serves as last resort. We present a novel strategy to cover large denuded wounds using the degloved skin as meshed full thickness skin grafts in two cases. 

## CASE REPORT

We present two male patients at the ages of 63 and 38 years with extensive degloving injuries of forearm and hands after being dragged by a train. The first case is a 63-year-old patient who was delivered to our hospital after a suicidal attempt, trying to amputate both his forearms by a train. This patient was treated medically for arterial hypertension and used antidepressant medication on an outpatient basis. On admission, his vital signs were stable; he was not intubated. After CT scanning and exclusion of other injuries than the forearms and hands, the patient was brought to the operating room. Fractures of metacarpal bones and phalanges were reduced and osteosynthesis using mini plates was performed. 

The 5^th^ ray on the right hand was amputated at the carpus; hypothenar muscles still being present. Because of the extensive degloving injury of the left forearm and hand, we decided on resecting the degloved skin at the level, where the skin was still attached to the forearm fascia. Then, the skin was cleansed mechanically and by rinsing it several times in betadine and polyhexanide solution. Afterwards, the skin was defatted and meshed using a Brennen Mesher mesh graft 1:3. Full thickness skin mesh graft was applied onto the defects. 

On the right hand, skin was cleansed mechanically and several times by betadine and polyhexanide solution, defatted to be used as full thickness skin graft and sutured back into place. Thereby, a full coverage of the degloving defects on both hands and arms was achieved. Then, vacuum assisted closure devices (VAC®) were attached for five days at 80 mm HG continuously. The patient received cefuroxime and metronidazole for 7 days. On day five, full thickness skin grafts were attached over 90%. The epidermis of full thickness skin graft on the right hand over the palm showed necrosis. Consequently, the full thickness graft was de-epithelialized and a split thickness skin graft was successfully used for defect coverage. The remaining defect over hypothenar and the forearm was covered using split thickness skin grafts from the lateral thigh, meshed 1:3. 

**Fig. 1:**
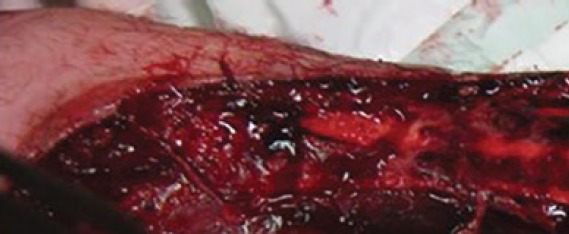
Second patient, presenting severe degloving injury of the left forearm and the right hand was not injured

The second patient was a 38 years old male, who was also treated for arterial hypertension and was who tripped on the platform besides the running train. He was admitted in a sedated, intubated and ventilated state by ambulance. Immediate investigation and CT scan revealed a large degloving injury of his left hand up to metacarpophalangeal joint and forearm up to the level proximal of the elbow. The little finger was completely degloved starting from the proximal phalanx. The distal phalanx of the left ring finger was amputated at the distal interphalangeal joint, and the tip of the middle finger was partially degloved. In addition, an undisplaced transverse fracture of the first metacarpal bone was present (Figure 1). 

The little finger was degloving amputated at the distal interphalangeal joint, the ring finger was amputated at the middle of the distal phalanx. In the operating room degloved skin was amputated at the level of attachment to the underlying fascia, cleansed mechanically and rinsed several times using betadine and polyhexanide solution. Then resected skin was treated as before. The little finger was amputated at the metacarpophalangeal joint and the ring finger at the distal interphalangeal joint. 

**Fig. 2:**
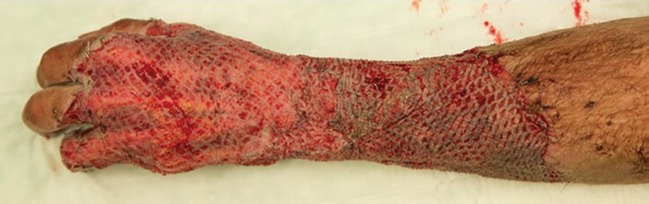
After five days of VAC® treatment of transplanted meshed full thickness (forearm) and split thickness skin (at the dorsum of the hand and fingers) grafts. The difference in the skin quality between meshed split thickness and full thickness skin grafts is visible

The transverse fracture of the first left metacarpal bone was reduced under x-ray control and plated using a mini-plate. Then, the meshed full thickness graft was wrapped around the degloved forearm and hand and hold in situ by skin staples. Thereby the entire degloving defect except the hypothenar could be covered. A VAC® was applied for five days; i.v. antibiotic treatment was installed as before. After five days, full thickness skin grafts were attached over 70% (Figure 2). 

**Fig. 3:**
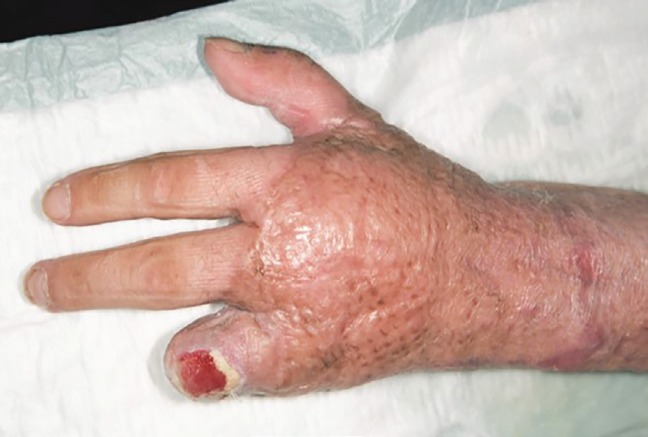
Four months after the first operation and 3 weeks of intense hand physiotherapy. On the palmar aspect, the distal forearm and the palm of the left hand are covered by a thinned ALTP flap. The difference in skin quality and shrinking of the epithelialized meshed full thickness and split thickness skin grafts is visible

The remaining defects at the hypothenar and on the distal forearm with partially uncovered ulnar artery and nerve were covered using a free supercharged ALTP flap from the contralateral side. Because of a scar contracture and posttraumatic arthrosis of the proximal interphalangeal joint of the ring finger, we performed an arthrodesis of the PIP IV joint of the left (Figure 3). 

Both patients showed fully covered defects. The degloving injuries were entirely covered; however, full thickness skin grafts had a tendency to shrink about estimated 15%, whereas the split thickness skin grafts shrank to about 30%. Therefore, both patients were sent to physiotherapy on an outpatient basis and were advised to cream the grafted skin on a daily basis. However, the first patient did not return to our outpatient clinic. The second patient had contractures at the wrist level of the left hand and admitted non-compliance, returning to our outpatient clinic two months later. After three months of intense hand physiotherapy, the patient demonstrated fist making and extension of the injured hand comparable to the contralateral side.

## DISCUSSION

Although abrasion-degloving injuries can be present to any part of the body, lower limb degloving injuries are the most common ones.^7^ However, degloving of the heels, upper limbs, or the scalp can lead to severe blood loss and shock.^2^^,^^11^^,^^12^ Especially degloving injuries in children require specialized complex surgical reconstruction to maintain function.^13^ Degloving of the forearm usually occurs at the level of the subcutaneous tissue, leaving the fascia and the muscles of the forearm intact. At the palm of the hand, the plane of separation is also the subcutaneous level. 

Palmar fascia, digital nerves and vessels are well protected along with the flexor tendons and the lumbrical muscles. At the dorsum of the hand the plane of separation is also at the subcutaneous level, leaving the extensor tendons exposed. However, an injury of the fascia covering the interossei muscles is rare. The skin of the fingers degloves at the subcutaneous level. Flexor and extensor tendons are usually intact, including the neurovascular bundles, leaving the function virtually intact.^2^ There are some classification systems of degloving injuries discussed in literature. 

Arnez *et al.* classified the patterns of abrasion or degloving injuries according to their depth and extent: limited abrasion –avulsion injuries, non-circumferential-, circumferential single-plane- and circumferential multi-plane-degloving injuries. In addition, they are classified as open or closed injuries, so eight patterns should be kept in mind.^14^ Ju *et al. *have reported on 41 cases of degloving injuries of the hand and based on these constructed a classification system of the hand. This system is based on the degree of the injury on the hand. 

The suggested treatments of the authors range from replantation over complex replantation combined with second-toe transplantation and skin flaps from the dorsum of the feet to reconstruction with thumb flaps containing dorsal skin.^15^ Yan *et al.* have evaluated 129 skin avulsion injuries of the lower extremities from 2002 to 2011. They have classified the degloving in pure degloving, degloving injuries with involvement of deep soft tissue and degloving injuries with long-bone fractures.^7^

Although the best classification system is still being discussed in literature, principles of treatment remain the same. Preserve as much tissue and length of denuded bone as possible by coverage with primary surgical treatment by using good quality skin and to start early mobilization to preserve function.^2^^,^^5^ Here the avulsed skin has been described used as a source for split thickness or full thickness skin graft. However, in these cases, when the degloved skin has been used as full-thickness skin graft, the graft was just fenestrated and then applied onto the denuded areas.^2^^,^^7^^,^^16^

In all cases these and other authors prefer the use of vacuum assisted wound closure devices to support the attachment of the replanted skin.^7^^,^^17^ Alternatively, in case of largely destroyed degloved skin, microsurgical reconstruction by use of anterolateral thigh flaps or latissimus dorsi flaps have been promoted.^9^^,^^10^ However, most of these procedures require expertise in microvascular surgery and very often, secondary surgical procedures cannot be avoided such as thinning of the flaps.^2^ Alternative suggested pedicled flaps are the groin flap, abdominal flap, combined groin and abdominal flap as a bilobed flap, or abdominal quadrant flap. In extensive degloving injuries of the entire palm and dorsum of the hand, abdominal pocketing is a useful procedure. However, also these flaps need secondary surgical procedures to achieve acceptable function of the injured hand and forearm.^2^

Additionally, when parts of the flaps do not heal, the denuded defects must be covered. Here often split thickness skin grafts are used.^2^^,^^7^ The known disadvantage is shrinking and scarring of the grafts, more than full thickness skin grafts.^2^^,^^5^ A new alternative opened when acellular dermal matrices combined with split thickness skin grafts where used.^8^^,^^18^ Graham *et al. *described the successful use of Integra® on ten patients with degloving injuries of areas between 500 – 1000 cm².^8^ Iorio *et al.* have performed a literature research and found 432 patients treated. The use of the dermal regeneration template is described as a simple technique to achieve durable tissue coverage.^18^ Other authors report on the use of small-intestine submucosa as a dermal matrix replacement.^19^

However, the treatment with Integra® relies on adequate vascular supply of the soft tissue bed, eradication of infection, off-loading and/or immobilization and the treatment with small-intestine submucosa required serial debridements.^18^^,^^19^ Additionally, the use of dermal regeneration templates is expensive and not every hospital can afford treatment using this treatment.^18^ We describe the use of degloved skin as a meshed full thickness skin graft. Although a very simple technique, the meshed full thickness skin graft covered large areas of the denuded wounds. Even smaller parts of degloved skin were usable as mesh graft and could be successfully applied onto defects for coverage. 

However, we also observed shrinking of the grafts, although not as much as split thickness skin grafts, in agreement with literature. To avoid functional deficits, early active and passive physiotherapy combined with adequate pain management is recommended. In addition, regular skin care during the time of reconvalescence not only improves the quality of the skin but also supports wound healing and shortens the time of healing. Additional treatment with compressive garments also supports skin flexibility and can help avoiding the formation of keloids.^20^

Our patient gained near to normal range of motion by continuous physiotherapy and the use of compressive garments. We present successful application of meshed full thickness skin grafts in large defect coverage made from the degloved skin. When using this technique, adjuvant procedures such as physiotherapy, standardized scar treatment, orthesis, and compression garments are recommended to achieve a good functional result. Therefore, we showed that the meshed full thickness skin graft technique could be a useful part of the armamentarium of plastic and trauma surgeons.
